# Peptide Vaccines for Pediatric High-Grade Glioma and Diffuse Midline Glioma: Current Progress and Future Perspectives

**DOI:** 10.3390/vaccines13121215

**Published:** 2025-11-30

**Authors:** Aron K. Mebrahtu, Vatsal Jain, Eliese M. Moelker, Alexandra M. Hoyt-Miggelbrink, Katayoun Ayasoufi, Eric M. Thompson

**Affiliations:** 1 School of Medicine, Duke University, Durham, NC 27710, USA; aron.mebrahtu@duke.edu; 2 Department of Neurosurgery, Duke University, Durham, NC 27710, USA; 3 The Preston Robert Tisch Brain Tumor Center, Duke University, Durham, NC 27710, USA; 4 Department of Neurological Surgery, Washington University, St. Louis, MO 63110, USA

**Keywords:** pediatric high-grade glioma, diffuse midline glioma (DMG), peptide vaccine, immunotherapy, H3.3K27M, CMV pp65, pseudo progression (PsPD), poly-ICLC, temozolomide

## Abstract

High-grade gliomas (HGGs) and diffuse midline gliomas (DMGs) in pediatric patients carry a poor prognosis, necessitating the rapid development of novel therapies. Peptide vaccines represent a safe, repeatable, and rational immunotherapeutic modality aimed at inducing potent, tumor-specific T-cell responses. In this review, we define the scope of current progress by arguing that immunogenicity in children with HGG/DMG hinges on three factors: appropriate antigen class (neoantigen vs. TAA), the use of potent immunoadjuvants, and successful navigation of immune suppression. To address the gap between biological promise and clinical reality, we analyze clinical trials targeting shared tumor-associated antigens (e.g., CMV pp65, Survivin) and specific shared neoantigens (H3.3K27M). Crucially, we highlight pivotal data from the PNOC007 trial, where the magnitude of H3.3K27M-specific T-cell expansion correlated directly with significantly longer overall survival (OS), establishing a causal link between pharmacodynamics and clinical benefit. However, the unique challenges of the immunosuppressive tumor microenvironment and the detrimental effect of necessary corticosteroids remain paramount barriers. Future success relies on multi-modal combination strategies, the development of next-generation personalized neoantigen vaccines, and the application of advanced neuroimaging to accurately assess treatment response.

## 1. Introduction

High-grade gliomas (HGGs) represent a formidable challenge in pediatric neuro-oncology. These are highly malignant, fast-growing tumors that arise from glial precursor or progenitor cells in the brain or spinal system. Pediatric HGGs typically present de novo, infiltrate, eloquent CNS tissue and resist cytotoxic therapy [1]. The World Health Organization’s (WHO’s) 2021 CNS Tumor Classification recognizes pediatric gliomas as a distinct category, “Pediatric type diffuse high-grade gliomas” (PDHGGs), acknowledging their unique molecular and pathological features compared to adult gliomas [2]. This classification includes aggressive subtypes such as diffuse midline glioma (DMG, H3 K27-altered), a subcategory of DMG that grows in the pons region of the brain stem known as diffuse intrinsic pontine glioma (DIPG), and diffuse hemispheric glioma, H3 G34 mutant, which are characterized by specific, unique histone H3 mutations.

The prognosis for children diagnosed with these tumors is devastatingly poor. The five-year survival is less than 20% overall, and for some particularly aggressive subtypes, such as DMG, the two-year survival is often less than 10% [3,4], with median survival commonly 9–18 months. These grim outcomes highlight the urgent and significant unmet need for more effective therapies and strategies beyond the current standard of care.

Current standard-of-care treatment for pediatric HGG includes maximal resection (or frequent biopsy of DMG), radiation therapy, and often chemotherapy, but their combined effectiveness is severely limited by the aggressive biology of the tumors and their location within the CNS making them inoperable [5,6,7]. Focal radiation therapy is a standard option for children 3 years of age and older. However, its use is avoided in very young children to mitigate the risk of developmental brain injury. The role of chemotherapy is even more contentious. While drugs like temozolomide (TMZ) have shown some efficacy in some adults, they are not as effective in children and rarely achieve durable control; conventional chemotherapeutic agents often only elicit a low objective response rate, typically less than 20% [8]. Despite treatment, pediatric HGGs recur at a high rate. Overall survival in pediatric recurrent HGG is only about 5.6 months [9]. The limitations of all these therapies highlight a significant and fundamental therapeutic gap. Given the limited efficacy of these conventional modalities and the risk of long-term neurotoxicity, there is a critical necessity for a new treatment paradigm that moves beyond currently available cytotoxic agents. Immunotherapy offers a compelling alternative; however, unlike broad-spectrum immune checkpoint inhibitors, which have shown limited success in “cold” pediatric tumors, peptide vaccines offer a highly specific mechanism of action. By delivering defined antigenic sequences, peptide vaccines can precisely target tumor antigens while maintaining a favorable safety profile, thereby harnessing and amplifying the body’s own defense systems without the systemic toxicity associated with other therapies.

## 2. Materials and Methods

### 2.1. Search Strategy and Data Sources

A comprehensive literature search was conducted to identify interventional clinical trials, observational studies, and mechanistic/translational papers investigating peptide-based immunotherapy in pediatric and young adult neuro-oncology. We queried the following electronic databases: MEDLINE (Ovid/PubMed), Embase (Ovid), Web of Science Core Collection, Scopus, and Cochrane CENTRAL. To ensure the inclusion of ongoing and recently completed trials, we also searched clinical trial registries, including ClinicalTrials.gov and the WHO International Clinical Trials Registry Platform (ICTRP). Furthermore, the grey literature and relevant abstracts from major neuro-oncology and oncology conferences (SNO, ISPNO, ASCO, and AACR) were reviewed to capture emerging data not yet published in peer-reviewed journals.

### 2.2. Search Terms and Keywords

Search strategies utilized a combination of Medical Subject Headings (MeSH) and free-text keywords using Boolean logic. The search algorithms were designed to intersect three primary concepts: (1) population (e.g., pediatric, adolescent, young adult); (2) pathology (e.g., high-grade glioma, diffuse midline glioma/DIPG, medulloblastoma, ependymoma); and (3) intervention (e.g., peptide vaccine, synthetic long peptide, neoantigen vaccine). Specific queries also included terms for common adjuvants (e.g., poly-ICLC, Montanide) and known antigen targets (e.g., H3.3K27M, CMV pp65, Survivin, IL-13Rα2) to ensure the capture of trials identified by target rather than modality.

### 2.3. Eligibility Criteria and Study Selection

Studies were selected based on specific inclusion and exclusion criteria.

Inclusion Criteria: We included studies involving pediatric patients and patients with AYA (defined as age ≤ 39 years) diagnosed with HGG, DMG/DIPG, or relevant pediatric CNS tumors where peptide vaccines have been tested (e.g., medulloblastoma, ependymoma). The intervention was strictly limited to peptide vaccines (short class-I peptides and synthetic long peptides) utilizing standard adjuvant platforms (Montanide, poly-ICLC/Hiltonol) or preconditioning regimens (Td toxoid, TMZ lymphodepletion). We included interventional trials of any phase, observational/expanded access programs, and mechanistic papers reporting human immunogenicity data (e.g., ELISpot, dextramer/CyTOF analysis).

Exclusion Criteria: To maintain a focus on synthetic peptide immunogenicity, we excluded whole-cell vaccines, dendritic-cell (DC) vaccines (unless the study explicitly utilized peptide-pulsing and was directly comparable to synthetic platforms), CAR-T-cell therapy, checkpoint inhibitor monotherapy, and oncolytic virus therapy.

## 3. Mechanisms of Peptide-Based Cancer Immunotherapy

**Figure 1:**
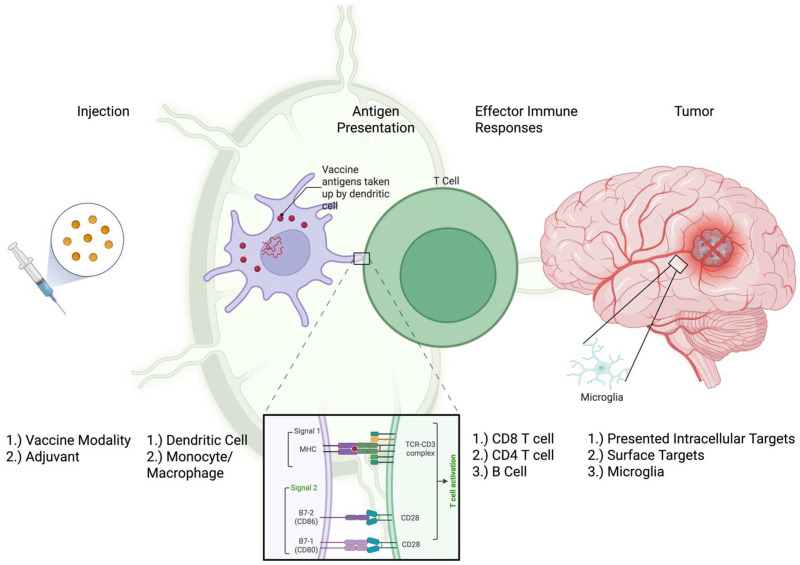
Possible mechanisms through which a peptide vaccine elicits immune responses in pediatric gliomas. Peptide(s) are delivered with an adjuvant/carrier. Then, antigen-presenting cells (APCs) take up peptides, mature, and present the peptide on MHC I/II. In the lymph node, APCs prime CD8^+^ and CD4^+^ T cells; CD4^+^ T helper cells can also facilitate the activation of B cells. Following this, activated lymphocytes (T and B cells) clonally expand and egress. Effector phase: effector cells and antibodies traffic to and infiltrate the CNS tumor (right panel). After this, cytotoxic T cells mediate perforin/granzyme-dependent killing and IFNγ signaling. Concurrently, plasma cells generate tumor-specific antibodies that can modulate tumor biology. This combined response promotes epitope spreading, which is mediated by CNS-resident APCs, including microglia. Responses are constrained by checkpoint pathways and a suppressive myeloid microenvironment. Created with BioRender (https://www.biorender.com/).

Peptide vaccines deliver defined antigenic sequences to prime tumor-specific T cells. Figure 1 shows the possible mechanisms through which a peptide vaccine elicits immune responses in pediatric gliomas. After intradermal/subcutaneous injection, peptides are typically captured and processed by antigen-presenting cells (APCs) including dendritic cells (DCs) and displayed as peptide–MHC complexes to T cells. Short class-I peptides (8–11 aa) directly load onto MHC-I and efficiently prime CD8^+^ responses but are HLA-restricted (e.g., HLA-A*02:01), narrowing eligibility. Synthetic long peptides (SLPs; ≥15–20 aa) must be processed by DCs and thereby present on both MHC-I and MHC-II, recruiting CD4^+^ help and broadening HLA coverage [10,11]. All of these processes require environmental cues to properly activate the T-cell response. In addition to presenting antigens, DCs also upregulate co-stimulatory molecules and release cytokines. The lack of these secondary signals is detrimental to T-cell activation and can result in impaired effector functions [12].

Because peptides alone are weakly immunogenic, pediatric trials pair antigen with immunoadjuvants that provide “danger signals” and sustained presentation. Currently, the platforms include:

### 3.1. Montanide ISA-51 + Poly-ICLC

Montanide ISA-51: Montanide ISA-51 is an oily depot that prolongs antigen availability to DCs at the subcutaneous injection site. This product is mixed with poly-ICLC (Hiltonol), which serves as a potent Toll-like receptor 3 (TLR3) and MDA5 agonist. Together, a strong “danger” signal” is initiated, including type-I interferon production, which “licenses” DCs for productive cross-priming (used in the Pollack and PNOC007 trials) [13,14,15].

### 3.2. Montanide ISA-51 + TMZ/Td Preconditioning

An alternative to poly-ICLC is short course of temozolomide (TMZ), which is delivered to induce transient lymphopenia, then followed by tetanus/diphtheria preconditioning at the injection site to enhance dendritic-cell migration [13]. While induction of lymphopenia appears counterproductive, this effect allows for significant expansion of primed antigen-specific T cells, enhancing the effect of the vaccine [14]. This strategy shapes a permissive priming milieu without the poly-ICLC and its associated downsides [10,11]. These downsides primarily include significant inflammatory reactions, such as common injection-site reactions [15] and symptomatic pseudoprogression (PsPD) that can require immune-suppressive steroid treatment [16,17].

Operationally, glucocorticoids such as dexamethasone are standard of care for cerebral edema, but they are, in turn, potent immune suppressors that blunt DC activation and T-cell expansion [18,19,20]. Therefore, steroid sparing at priming is a mandatory design necessity that recurs across pediatric studies.

### 3.3. Antigen Selection: TAAs vs. TSAs and Rationale for Multi-Antigen Targeting

The selection of an appropriate antigen is critical for the success of any vaccine. As summarized in Table 1, these antigens can be broadly categorized into two groups: tumor-associated antigens (TAAs) and tumor-specific neoantigens (TSAs).

Tumor-associated antigens (TAAs): TAAs are proteins that are overexpressed by tumor cells but also present in low levels in healthy tissues [21]. Examples of TAAs targeted in pediatric glioma vaccines include the cytomegalovirus (CMV) pp65 antigen and glioma-associated antigens (GAAs) such as EphA2, IL13RA2, and Survivin. While these antigens are often accessible and abundant targets, their expression in normal cells has the potential to lead to a state of immune tolerance. Central tolerance leads to deletion of self-antigen reactive T cells, which recognize self-antigen-MHC complexes in the thymus [25]. As such, the immune system likely recognizes EphA2, IL13RA2, and Survivin as “self-antigens,” resulting in weak or no T-cell responses against these antigens due to thymic deletion of high-affinity T-cells or low-affinity T-cell receptor (TCR) binding [21]. In contrast, anti-CMV responses in most humans are strong, as these antigens are virus-derived and hence foreign antigens. However, due to the nature of CMV and its ability to modulate immune responses during latency, it can induce peripheral immune tolerance. Such an inherent limitation necessitates the use of potent adjuvants to overcome tolerance and elicit a robust immune response.

Tumor-specific neoantigens (TSAs): In contrast, TSAs are de novo antigens that arise from unique, tumor-specific mutations and are not expressed in normal, healthy tissue. The H3.3K27M mutation, which is pathognomonic for a large proportion of diffuse midline gliomas, is a shared neoantigen that serves as an ideal vaccine target due to its high specificity and expression in nearly all tumor cells [21,22]. A vaccine targeting this mutation may be less likely to induce off-target effects and may not result in immune tolerance. While targeting these antigens is possible, we must note that these antigens still have a limitation, as they closely resemble their “self-antigen” counterpart as opposed to a true foreign antigen and as such elicit a weak if any immune response.

Beyond shared neoantigens, the field is advancing toward personalized neoantigen vaccines, which are custom designed based on a patient’s unique tumor mutations [26].

Such an antigen could be a variant of self (similar to the H3.3K27M mutation), a true viral antigen (including CMV’s PP65), or even against endogenous retroviruses [27]. This approach directly addresses the problem of intratumoral heterogeneity, where a single biopsy may not capture all relevant antigens, and aims to generate a highly specific and potent anti-tumor response. This approach will also take into account the host MHC and can incorporate long-peptide design (that can be chopped by host APCs to fit appropriate MHCs) or predicted host–MHC binding interactions.

One of the most critical challenges in glioma vaccine design is the selection of antigens that are (1.) consistently expressed and not hindered by tumor heterogeneity and (2.) can overcome central and peripheral immune tolerance. Gliomas exhibit significant tumor heterogeneity and clonal diversity, meaning not all cells within a single tumor express the same antigens uniformly. A monovalent vaccine that targets a single antigen risks failure if the tumor undergoes “immune-editing” and ceases to express the targeted protein to escape immune surveillance. Therefore, multipeptide or polyepitope vaccines, such as the cocktail targeting EphA2, IL-13Rα, and Survivin, are employed to provide a robust and more resilient immune response.

### 3.4. Clinical Evidence

#### 3.4.1. Shared TAA Short-Peptide + Montanide ISA51 + Poly-ICLC

As evidenced in Table 2, this platform uses short, HLA-A*02:01-restricted peptides targeting EphA2, IL-13Rα, and Survivin, combined with Montanide ISA-51 and poly-ICLC adjuvant.

Pollack et al. [13] (newly diagnosed HGG/DIPG): A pilot study (NCT01130077) in post-radiotherapy children found the regimen safe and feasible. It was highly immunogenic, with 13/21 evaluable patients (>50%) mounting ELISpot responses to at least one glioma-associated antigen. The incidence of symptomatic pseudo progression (PsPD) was ≈19% (5/26), which was often associated with longer survival [16].

Pollack et al. [14,28] (recurrent HGG): A follow-up study in children with recurrent HGG confirmed safety and high immunogenicity, with 9/10 (90%) mounting antigen-specific CD8^+^ responses by ELISpot. The trial reported one patient with a durable partial response, lasting over 39 months [17,28].

**Table 2:** Results of peptide vaccine clinical trials.

*Trial (First Author, Year)*	*ID*	*N*	*Tumor Type(s)*	*Patient Population*	*Key Immunogenicity Findings*	*Key Clinical Outcomes (PFS/OS)*
*Pollack et al., 2014* [16]	NCT01130077	26	HGG, brainstem glioma (BSG)	Newly diagnosed	Showed evidence of immunological activity	Evidence of clinical activity; some patients had sustained stable disease
*Pollack et al., 2016* [17]	NCT01130077	12	HGG	Recurrent	Immune response in 90% of evaluable patients	Median PFS 4.1 months, OS 12.9 months; one partial response for >39 months
*Thompson et al., 2025* [24]	NCT03299309	42	HGG, medulloblastoma	Recurrent	Successfully induced a significant increase in pp65-reactive T- cells	Median PFS 2.5 months, OS 6.4 months; 12-month OS 26.6%
*Mueller et al., 2020* [15]	NCT02960230	19	Diffuse midline glioma	Newly diagnosed	H3.3K27M-specific CD8T-cell expansion was detected	Responders had a median OS of 16.1 months vs. 9.8 months for non-responders

#### 3.4.2. Neoantigen Short-Peptide + Montanide + Poly-ICLC

The PNOC007 Phase I/II trial (NCT02960230) targeted the shared H3.3K27M neoantigen in newly diagnosed patients with DMG/DIPG (HLA-A*02:01+) post-radiation [15].

Key finding: The vaccine was well-tolerated, with injection-site reactions being the most common adverse event. Using advanced mass cytometry (CyTOF) and dextramer assays, the study successfully demonstrated that the vaccine induced an expansion of H3.3K27M-reactive CD8 + T cells in the peripheral blood.

Clinical correlation: Patients who developed an H3.3K27M-specific CD8 + T-cell expansion had a significantly longer median overall survival (OS) of 16.1 months compared to 9.8 months for non-responders.

Impact of steroids: This trial explicitly identified that immediate pretreatment dexamethasone administration was inversely associated with T-cell responses and a shorter OS. Lower baseline levels of monocytic myeloid-derived suppressor cells (M-MDSCs) (CD33+ CD11b+ CD14+ HLA-DR^lo^) were also associated with longer OS/PFS.

#### 3.4.3. HLA-Agnostic SLP + Montanide with TMZ/Td Preconditioning (PEP-CMV)

The PEP-CMV vaccine targets the CMV pp65 viral antigen using a synthetic long peptide (SLP) format (26 mer), designed to be HLA-agnostic (i.e., works irrespective of patient HLA variant). The Phase I trial (NCT03299309) was conducted in children and young adults with recurrent HGG or medulloblastoma [24].

Immunogenicity: The trial confirmed the platform was safe and highly immunogenic. ELISpot assays demonstrated significant expansion of pp65-specific T cells in 16 of 21 evaluable patients (76.2%). While the SLP design was intended to recruit both CD4+ and CD8+ responses, detailed immunophenotyping revealed that survival benefit specifically correlated with an increased percentage of terminally differentiated effector memory (TEMRA) CD8+ T cells.

Clinical outcomes: Clinical outcomes were modest in the recurrent setting (median PFS of 2.5 months and median OS of 6.5 months), with the 12-month OS rate of 28% noted as a promising signal.

Immune correlate: Detailed immunophenotyping identified three key prognostic markers. Improved progression-free survival (PFS) was significantly associated with higher baseline counts of naïve CD8+ T cells (*p* = 0.006) and lower frequencies of CD56 Bright CD16 Dim NK cells (*p* = 0.029). Additionally, an expansion of terminally differentiated effector memory (TEMRA) CD8+ T cells following vaccination tracked with longer subsequent survival.

## 4. Response Assessment and Immune Analysis

### 4.1. Response Assessment and Imaging Pitfalls

Pseudoprogression: A major diagnostic challenge in glioma immunotherapy is the phenomenon of pseudoprogression (PsPD) [29]. PsPD is a temporary increase in tumor size on imaging, caused by an inflammatory influx of immune cells and is a positive sign of an active immune response. However, because PsPD mimics true tumor growth on standard MRI, it often meets the radiographic criteria for “progressive disease” (e.g., RANO criteria). This creates a risk that patients will be erroneously removed from a potentially beneficial therapy due to the appearance of treatment failure. The trials used with the Pittsburgh platform (Pollack et al.) established a protocolized management plan for PsPD: hold the vaccine/poly-ICLC administration; treat the resulting edema with dexamethasone; re-image and resume vaccination if steroids can be successfully weaned [16,17].

To accurately mange the ambiguity and clinical difficulties of PsPD, trials are integrating advanced neuroimaging techniques as non-invasive biomarkers of treatment response:

Parametric response mapping of apparent diffusion coefficient (PRM-ADC): This specific MRI technique can help clinicians distinguish between true tumor growth and inflammatory swelling [30].

Magnetic resonance spectroscopy (MRS): MRS measures the levels of key metabolites (e.g., N-acetylaspartate, choline, myoinositol [mI]) and is being investigated to monitor disease status in patients with DIPG [31].

### 4.2. Immune Biomarkers and Mechanistic Correlates

Developing reliable immune endpoints is essential for predicting patient benefit and refining vaccine design.

Peripheral pharmacodynamics endpoint immunogenicity is monitored via peripheral blood assays that measure the activation and expansion of antigen-specific T cells:

IFNγ ELISpot: The enzyme-linked immunospot (ELISpot) assay is widely used gold standard to evaluate an antigen-specific immune response. It quantifies the number of T cells that produce anti-tumor cytokine interferon-gamma (IFNγ) in response to the target antigen.

Dextramer and mass cytometry (CyTOF): Highly multiplexed techniques like dextramer staining and CyTOF mass cytometry (used in PNOC007) allow for the precise identification and phenotypic characterization of rare antigen-specific CD8 + T cells, linking T-cell expansion to improved survival.

Prognostic factors and suppressive axes: Clinical trials have established that baseline immune suppression sufficiently gates vaccine efficacy.

Dexamethasone and immunosuppressive responses by myeloid cells: The PNOC007 trial conclusively showed that immediate pretreatment dexamethasone administration was inversely associated with T-cell responses and shorter OS. The immune suppression was quantified using high-dimensional mass cytometry (CyTOF) profiling of peripheral blood mononuclear cells (PBMCs). This analysis identified a specific population of monocytic myeloid-derived suppressor cells (m-MDSCs), immunophenotypically defined as CD33+ CD11b+ CD14+ HLA-DR^lo^. Lower baseline levels of these specific suppressive cells were associated with longer OS and PFS [15]. Additionally, clinical data confirmed that immediate pretreatment dexamethasone administration was inversely associated with the magnitude of H3.3K27M-specific T-cell expansion.

Regulatory T cells (Tregs) and NK cells: The PEP-CMV trial identified that higher pretreatment Treg percentages correlated with worse PFS (hazard ratio (HR) 1.79 (95% CI: 1.03, 3.11), while trends in natural killer (NK) cell subsets (specifically CD56 Bright CD16 Dim/+) were also associated with survival [24]. Although responses to treatment (anti-CMV immune responses) did not predict or correlate with survival measures, the trial did convey a remarkable result: two long-term survivors who remain progression-free 5 years post-diagnosis of recurrent HGG. Analysis of the PRiME clinical trial data revealed that, compared to healthy controls, all patients with HGG had significantly higher numbers of only one immune cell type, one of five populations of NK cells: CD56 Bright CD16 Dim NK cells. These cells are thought to predict poorer survival in the literature; albeit this has only been described in a few contexts such as melanoma [32]. These findings underscore the power of combined innate and adaptive immune crosstalk.

These results translate directly into critical design implications for future trials: they must pre-specify antigen-specific T-cell expansion as a key pharmacodynamic endpoint, enforce strict steroid-sparing protocols (or steroid caps) during the priming phase, and rationally incorporate myeloid- or NK-targeting combination strategies to counter the profound immunosuppression observed in these cells. For example, the ongoing CONNECT1906 Phase II study (NCT05096481) will prospectively evaluate NK-cell subsets as prognostic biomarkers for the PEP-CMV platform, setting the stage for subsequent innate immune-modulating combination trials.

## 5. Future Directions

There are numerous obstacles to consider in peptide vaccine immunotherapy that will continue to be addressed in the design of these agents. The immunosuppressive microenvironment (TME): the glioma TME is inherently hostile (“immune cold”), characterized by a lack of tumor-infiltrating lymphocytes (TILs) and an abundance of immunosuppressive elements (myeloid cells, T regulatory cells, etc.) [33]. The blood–brain barrier (BBB) and blood–brain–tumor barrier (BBTB) serve as physical obstacles, impeding the efficacy of larger molecules including drug conjugates. Timing post-radiation therapy/finding the sweet spot: the post-RT period is an immunologically opportune “sweet spot” because RT induces immunogenic cell death, BBB permeability, release of neoantigens, and upregulated MHC-I expression, but the same period carries the risk of severe edema, requiring high-dose steroids [34].

Combination strategies: The collective evidence strongly suggests that peptide vaccines are most effective as the cornerstone of a broader multi-modal strategy. The transition to immune-immune combinations is now underway. For example, the PRiME (PEP-CMV + Nivolumab for Newly Diagnosed Diffuse Midline Glioma/High-grade Glioma and Recurrent Diffuse Midline Glioma/High-grade Glioma, Medulloblastoma, and Ependymoma) clinical platform is currently evaluating the combination of the PEP-CMV vaccine with the checkpoint inhibitor nivolumab (PRiME II; NCT06639607) to prevent T-cell exhaustion [35]. Similarly, the Intercept H3 trial (NCT04808245) is investigating the combination of an H3.3K27M-specific vaccine with nivolumab in patients with DMG, testing the hypothesis that checkpoint blockade can amplify vaccine-induced responses in this “cold” tumor type [36].

Peptide vaccine + immune checkpoint inhibitors (ICIs): Vaccines “prime” the immune system, while ICIs release the “brakes” put on these T cells by the immunosuppressive TME. The PNOC007 trial’s findings regarding the steroid/MDSC headwind further motivate combination with checkpoint or myeloid-modulating strategies.

Synergy with CAR-T-cell therapy: Peptide vaccines “prime” the immune system, while CAR-T cells deliver a focused, engineered cytotoxic attack against tumor cells. Reviews of glioma immunotherapy identify both peptide vaccines (e.g., rindopepimut, IMA950, IDH1) and CAR-T cells as promising combination options that may overcome antigen escape and limited immune persistence [37,38]. Preclinical insights further suggest that vaccine-induced priming can enhance CAR-T cell expansion, trafficking, and durability, while promoting T-cell recruitment and antigen spreading [39].

Next-generation and personalized vaccines: The future involves moving beyond shared antigens to highly tailored, personalized neoantigen sets, custom-designed based on an individual patient’s unique tumor mutations. This approach targets antigens that are “truly tumor-specific,” maximizing the potential for an anti-tolerance immune response. An active UCLA trial targeting the H3 G34 mutant is an example of this highly specific, next-generation approach (NCT06342908) [23].

Beyond this, there are other examples (see Table 3 for a list of ongoing trials):

The “warehouse” concept, as demonstrated by the GAPVAC-101 trial, utilizes a pre-manufactured library of off-the-shelf peptides (TAAs and shared neoantigens) matched to the patient’s specific tumor profile. This allows for personalization without the long manufacturing timelines of de novo synthesis [40]. Moving a step further, fully personalized neoantigen vaccines targeting unique somatic mutations have shown feasibility in adult glioblastoma. Keskin et al. (NeoVax) demonstrated that a personalized multi-epitope neoantigen vaccine could generate circulating antigen-specific T cells that successfully trafficked to the intracranial tumor [41]. In parallel, fixed multipeptide vaccines like IMA950, which targets a fixed set of glioblastoma-associated antigens (e.g., BCA, CSPG4), are also being explored in combination with checkpoint inhibitors [42,43].

IDH1(R132H) peptide vaccine (NCT02454634): The success of the IDH1(R132H) peptide vaccine in IDH-mutant gliomas provides a template for personalized neoantigen targeting. This Phase I trial demonstrated that the vaccine was safe, with adverse events restricted to Grade 1 [44].

Crucially, immune responses were observed in 93.3% of patients across multiple MHC alleles. The trial showed that patients with an IDH1(R132H)-specific immune response had an excellent two-year progression-free rate of 0.82, and this vaccine-induced response was associated with the presence of T-helper cells at the tumor site, thereby altering the tumor microenvironment. The high frequency of pseudoprogression observed further highlighted the active intratumoral inflammatory reactions induced by the vaccine.

mRNA vaccine platforms: The development of mRNA-based vaccines is an emerging hotspot. An experimental mRNA vaccine has shown remarkable success in mouse models by broadly “revving up the immune system itself,” potentially leading to a universal cancer vaccine approach [45].

**Table 3:** Ongoing and upcoming peptide vaccine trials in pediatric HGG/DMG.

*Trial ID*	*Phase*	*Name/Target*	*Patient Population*	*Primary Intervention*
*NCT05096481* [46]	II	CONNECT 1906 (PEP-CMV)	Newly diagnosed DMG/HGG, recurrent medulloblastoma	CMV pp65 SLP
*NCT06639607* [36]	I/II	PRiME II (PEP-CMV)	Newly diagnosed DMG/HGG	CMV pp65 SLP + nivolumab
*NCT05096481* [47]	I	Intercept H3	H3K27M+ DMG/HGG	H3.3K27M vaccine + nivolumab
*NCT06342908* [23]	I	Neoantigen- targeted ppDC	H3 G34 mutant glioma	Personalized neoantigen
*NCT04943848* [35]	I	DIPGVax	DIPG/DMG	H3.3K27M peptide + checkpoint
*NCT04749641* [48]	I	H3.3-K27M	HGG/DMG	IDH1 peptide (Survivin/poly-ICLC)

## 6. Conclusions

Peptide vaccines are a foundationally important and safe therapeutic strategy in pediatric HGG/DMG. Clinical data, particularly from the PEP-CMV [24], PNOC007 [15], and Pollack et al. [16,17] trials, confirm that a vaccine can induce a tumor-specific immune response in children that correlates directly with prolonged survival, thereby validating the therapeutic concept. However, efficacy is currently limited by the profoundly immunosuppressive TME, the physical barrier of the BBB, and the clinical requirement for immune-suppressive steroids. The next wave of trials will focus on overcoming these challenges through mandatory steroid-sparing protocols, the design of personalized neoantigen vaccines, and the rational combination of vaccines with checkpoint inhibitors or TME-modulating agents. The ultimate goal is to move beyond the poor OS of conventional past therapies and to further establish immunotherapy as a powerful therapeutic tool in the treatment of children with malignant brain tumors.

## Figures and Tables

**Table 1:** Comparison of peptide vaccines in clinical trials.

Antigen Class	Key Target(s)	Description	Immunogenicity Challenges
Shared neoantigen (TSA)	H3.3K27M (pathognomonic for DMG/DIPG) [15,21,22]. H3 G34-mutant (targeted in new Trials [23].	These arise from tumor-specific mutational burden; not expressed in normal tissue.	Low mutational burden in DMG requires precise molecular screening to confirm target presence.
Tumor- associated antigens (TAAs)	EphA2, IL-13Rα2, Survivin (frequently co-expressed in pediatric gliomas) [16,17,21].	These are overexpressed by tumor cells but are also present (in low levels) in healthy tissue.	Risk of immune tolerance (“self-antigen”) requires potent adjuvants.
Viral Antigen	CMV pp65 (widely expressed on most HGG and medulloblastoma tumors) [24]	These are viral proteins frequently expressed by a large fraction of tumor cells in HGG/DIPG/ medulloblastoma.	Novel and tests the concept of viral immunogenicity being leveraged against tumor.
